# Pleiotropic action of CpG-ODN on endothelium and macrophages attenuates angiogenesis through distinct pathways

**DOI:** 10.1038/srep31873

**Published:** 2016-08-25

**Authors:** Jiahui Wu, Wenru Su, Michael B. Powner, Jian Liu, David A. Copland, Marcus Fruttiger, Paolo Madeddu, Andrew D. Dick, Lei Liu

**Affiliations:** 1 Academic Unit of Ophthalmology, School of Clinical Sciences, University of Bristol, UK; 2 State Key Laboratory of Ophthalmology, Zhongshan Ophthalmic Centre, Sun Yat-sen University, Guangzhou, China; 3 Centre for Clinic Immunology, Sun Yat-sen University Third Affiliated Hospital, Guangzhou, China; 4 UCL-Institute of Ophthalmology, University College London, London, UK; 5 Bristol Heart Institute, School of Clinical Sciences, University of Bristol, UK; 6 National Institute for Health Research (NIHR) Biomedical Research Centre, Moorfields Eye Hospital, London, UK.

## Abstract

There is an integral relationship between vascular cells and leukocytes in supporting healthy tissue homeostasis. Furthermore, activation of these two cellular components is key for tissue repair following injury. Toll-like receptors (TLRs) play a role in innate immunity defending the organism against infection, but their contribution to angiogenesis remains unclear. Here we used synthetic TLR9 agonists, cytosine-phosphate-guanosine oligodeoxynucleotides (CpG-ODN), to investigate the role of TLR9 in vascular pathophysiology and identify potential therapeutic translation. We demonstrate that CpG-ODN stimulates inflammation yet inhibits angiogenesis. Regulation of angiogenesis by CpG-ODN is pervasive and tissue non-specific. Further, we noted that synthetic CpG-ODN requires backbone phosphorothioate but not TLR9 activation to render and maintain endothelial stalk cells quiescent. CpG-ODN pre-treated endothelial cells enhance macrophage migration but restrain pericyte mobilisation. CpG-ODN attenuation of angiogenesis, however, remains TLR9-dependent, as inhibition is lost in TLR9 deficient mice. Additionally, CpG-ODNs induce an M1 macrophage phenotype that restricts angiogenesis. The effects mediated by CpG-ODNs can therefore modulate both endothelial cells and macrophages through distinct pathways, providing potential therapeutic application in ocular vascular disease.

Angiogenesis is critical to physiological homeostasis, maintaining tissue health and supporting repair processes, such as wound healing and tissue regeneration. On the other hand, pathological angiogenesis occurs in disease situations, such as diabetes and cancer. Therefore, pharmacological control of angiogenesis represents a valuable therapeutic target for a wide spectrum of conditions. The process consists of multiple events, including initial disruption of vascular integrity (detachment of mural cells and extracellular matrix disruption), followed by a productive stage (proliferation, migration, sprouting, and tubing of vascular endothelial cells) and final stabilisation (incorporation of vascular smooth muscle cells and pericytes) resulting in a functionally competent vascular network^1^,^2^. A spectrum of angiocrine factors and chemokines modulate the different stages of angiogenesis^3^,^4^. Furthermore, recruitment of inflammatory and immune cells occurs in parallel, modifying and amplifying the processes afforded by local vascular cells^5^. Macrophages contribute significantly within a multicellular environment to angiogenesis, in part through release of paracrine promoters of tissue repair, but also through pro-inflammatory factors^6^. For example, although not exclusively, M1 macrophage phenotype is considered pro-inflammatory, while M2 macrophages are associated with vascular healing^7^.

Toll-like receptors (TLRs) widely expressed in multiple tissues and cell types belong to the pattern recognition receptor family and function as part of the first line of defence in the innate immune system by detecting the presence of pathogens^8^. The roles of TLRs in inflammation have been reported in a large number of studies^9^,^10^. TLRs also regulate angiogenesis in a variety of inflammatory settings^11^. However, the TLRs are pleomorphic and functionally disparate, and the current study examined anti-angiogenic effects mediated by specific TLR9 agonists, cytosine-phosphate-guanosine oligodeoxynucleotides (CpG-ODNs). CpG-ODNs have been shown to inhibit suture-induced mouse corneal neovascularization^12^, microvessel formation and tumour growth^13^, but whether these anti-angiogenic effects extends to other angiogenesis-related diseases and can offer therapeutic potential has not been fully explored. The effects mediated by CpG-ODNs is dependent on the number and location of CpG motifs, but also the backbone structure and length of the nucleotides^14^. As the natural ODN backbone phosphodiester (PD) is easily degraded by nucleases^14^, most of the current synthetic CpG-ODNs used in tumour clinical trials are phosphorothioate (PS)-modified to enhance stability^14^,^15^,^16^. These chemically modified synthetic CpG-ODNs are divided into three classes (class A, B and C) based on their sequence backbones and location of CpG motifs with distinct biological responses^17^. TLR9 expression is localised to intracellular membranes and activated within the endolysosome. When unmethylated CpG motifs (derived from viral or bacterial DNA) is recognised by TLR9, activation engages adaptor protein MyD88 and NF-κB to initiate an innate inflammatory response^8^. CpG-ODNs are synthetic TLR9 agonists and have been exploited as adjuvants for anti-tumour and vaccine therapeutics. CpG-ODN activation of TLR9 induces anti-tumour immunity and suppresses angiogenesis in tumours^12^,^18^,^19^,^20^,^21^,^22^.

Following our initial findings^12^, the aim of this study was to refine our understanding of pathways utilised by the family of CpG-ODNs mediated anti-angiogenesis in ocular angiogenesis models. We demonstrate that the 3 classes of CpG-ODN can each inhibit angiogenic responses in the eye, and their addition to endothelial cells promotes macrophage trafficking but reduced pericytes mobilisation. Furthermore, we also show that suppression of endothelial cell activity by CpG-ODNs is dependent upon PS-modified backbone and is not TLR9-dependent. However, TLR9-dependent macrophage activation by CpG-ODNs was required to promote M1 phenotype and modulate angiogenesis through regulation of endothelial cell function.

## Results

### Three classes of CpG-ODNs suppress angiogenesis by differentially regulating expression of VEGF and sFlt1

**Figure 1:**
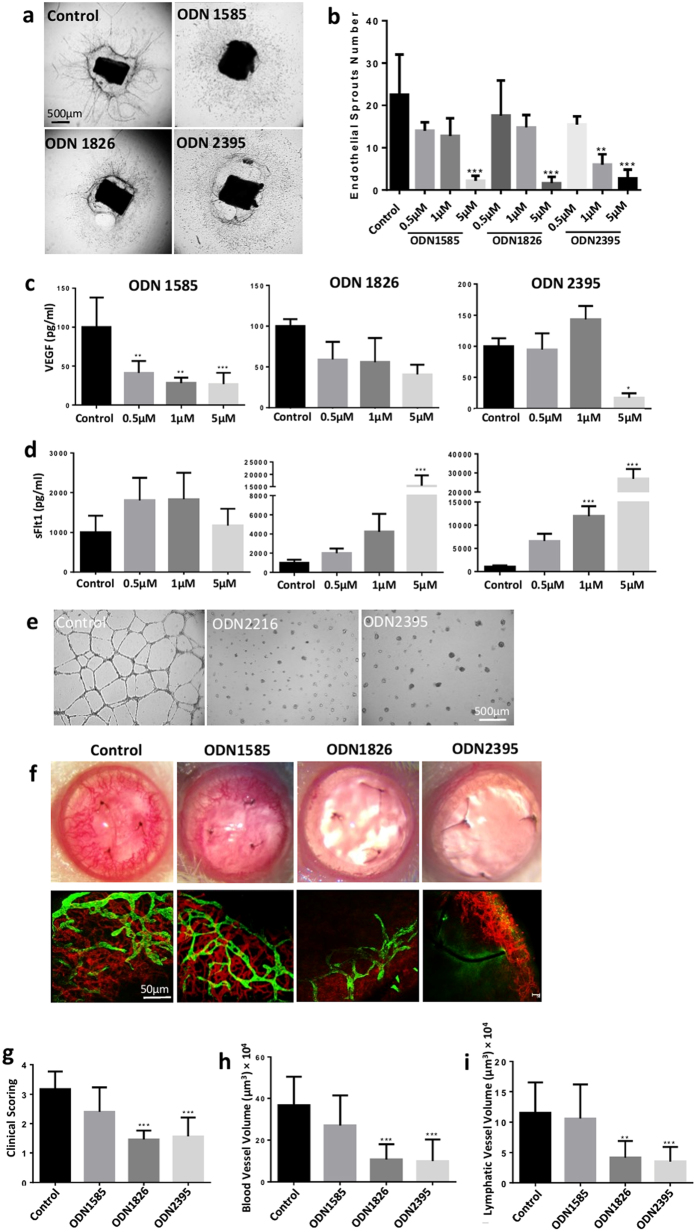
Three classes of CpG-ODNs suppress angiogenesis differentially by regulating expression of VEGF and sFlt1. (**a**) Representative phase-contrast photos of mouse aortic rings show the angiogenic inhibition by CpG-ODNs at day 7. Scale bar: 500 μm. Photos were taken under Wide field microscope (Leica DMI6000) with x5 magnification lens, each photo was composed of 4 images to represent one whole picture of each ring treated with 5 μM CpG-ODNs. (**b**) The sprout number was counted under Leica SP5-AOBS confocal laser scanning microscope (x5) after whole mount immunofluorescent staining with anti-CD31-APC (1:100, n = 15–18 per condition). All three classes of CpG-ODNs (class A: ODN1585; class B: ODN1826; class C: ODN2395) suppressed aortic ring sprouts compared to medium control at a dose-dependent manner. (**c,d**) The VEGF production in the supernatant of aortic rings was decreased by both class A and class C CpG-ODNs, while both class B and class C CpG-ODNs increased the sFlt1 production in supernatant tested by ELISA. (**e**) Class A and C CpG-ODNs completely inhibited the HUVECs tube formation compared to medium control after 24 hours (n = 12 per condition). Scale bar, 500 μm. (**f**) Upper row: Representative photos of suture-induced corneal angiogenesis model 7 days post sub-conjunctival administration of water or CpG-ODNs. Lower row: flat mount immunofluorescent staining of sutured cornea with anti-CD31 (red, 1:100) and anti-LYVE1 antibodies (green, 1:200) (n = 18–20 per condition). Image capture was performed with Leica SP5 microscope (x20). Scale bar, 50 μm. (**g–i**) Class B and class C but not class A CpG-ODN suppressed both hem- and lymph-angiogenesis in length and volume compared to vehicle control. Data represents means ± SD of relative values vs control from 3 independent experiments. *p < 0.05; **p < 0.005; ***p < 0.0005, statistical analysis was performed with one-way ANOVA with Dunn’s test.

Whilst the pro-inflammatory response induced by CpG-ODNs is recognised, these agents also modulate other functional effects, and we have previously demonstrated that ODN1826 can inhibit angiogenesis^12^. To expand these observations, we investigated whether this anti-angiogenic effect was ubiquitous to all 3 classes of CpG-ODN. Initially, mouse aortic rings were treated with three classes of murine CpG-ODN and their non-CpG-ODN controls (in which the CpG motifs were replaced by GpC). All three classes of CpG-ODN (class A: ODN1585; class B: ODN1826; class C: ODN2395) and their internal controls suppressed the formation of aortic endothelial sprouts in a dose-dependent manner (Fig. 1a,b and supplementary Fig. S1a,b). Assessment of VEGF and sFlt1 proteins in explant supernatants revealed differential expression of these factors depending on the class of ODN present. Class A at all tested concentrations and class C only at the high dose (5 μM) significantly decreased VEGF production (Fig. 1c). In contrast, class B and C at the high dose significantly increased sFlt1 production (Fig. 1d). Furthermore, in HUVEC tube formation assay, human class A (ODN2216) and class C (ODN2395) as well as their controls markedly suppressed HUVEC tube formation (Fig. 1e and Supplementary Fig. S1c).

Next, we tested all three classes of CpG-ODN and their non-CpG-ODN controls *in vivo* using the suture-induced corneal angiogenesis model. Class B and C inhibited corneal angiogenesis, showing a significant reduction in length and volume of blood and lymphatic vessels, whilst no effect was observed with class A or control ODN treatments (Fig. 1f–i and Supplementary Fig. S1d). Previously, no significant change in VEGF or sFlt-1 protein expression in cornea was detected following class B treatment^12^, and this was confirmed at the transcriptional level (Supplementary Fig. S1e). In summary, all three classes of CpG-ODN inhibited aortic vascular sprouting but only class B and C CpG-ODNs suppressed corneal angiogenesis. We therefore focused on these two classes in the following experiments. Increased sFlt-1 secretion (aortic ring assay) and *in vivo* suppression of corneal neovascularisation support current evidence that expression of sFlt-1 in the normal cornea regulates vascular growth^23^. Furthermore, class A failed to suppress corneal angiogenesis despite evidence of reduced VEGF expression, which suggests sFlt1 dominance as a pathway to suppress corneal angiogenesis.

### ODNs regulate angiogenesis in a backbone dependent but CpG motif independent manner

TLR9 is widely expressed in endosomes in many different cell types^11^, and activation of the TLR9 signalling pathway is dependent on binding CpG-ODNs. However, the downstream activity of CpG-ODN signalling is influenced by both the number of CpG-motifs and the structural backbone sequence^17^,^24^. In the current context, it was important to determine whether the CpG motif and structure were central to elicit the observed anti-angiogenic effects. To this end, ODNs were customised as described in Supplementary Table S1: PD-CpG-ODN and PS-CpG-ODN share the same sequence motif but with different backbones; while PS-CpG-ODN and PS-ApG-ODN share same backbone but an altered sequence, in which the cytosine is replaced with adenine in CpG motifs.

**Figure 2:**
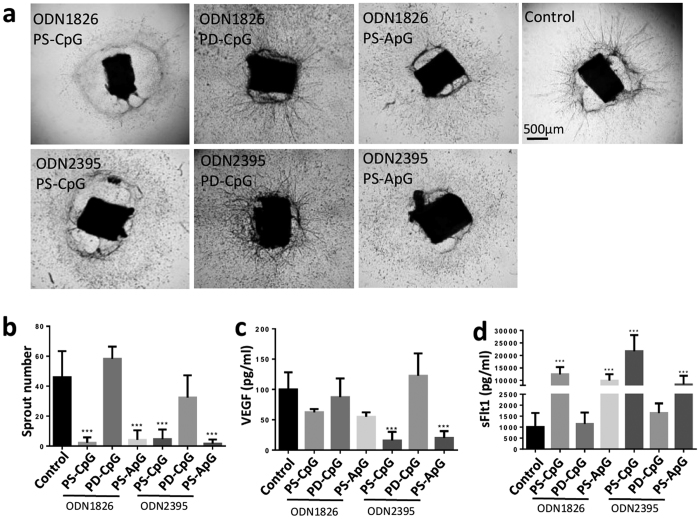
ODN regulates angiogenesis in backbone dependent but CpG motif independent manner. (**a,b**) Representative phase contrast photos of aortic rings, 7 days after stimulation with PS-CpG-ODNs, PD-CpG-ODNs, or PS-ApC-ODNs at the concentration of 5 μM (n = 18–23 per condition). Scale bar, 500 μm. Both PS-CpG-ODN and PS-ApG-ODN but not PD-CpG-ODN suppressed the aortic sprouts. (**c,d**) Both class B and C PS-CpG-ODNs and PS-ApG-ODNs increased sFlt1 production, whilst class C PS-CpG-ODNs and PS-ApG-ODNs decreased VEGF production measured by ELISA of the supernatant. Data represents means ± SD of relative values vs control from 3 independent experiments. *p < 0.05; **p < 0.005; ***p < 0.0005, statistical analysis was performed with one-way ANOVA with Dunn’s test.

When applied to aortic ring assay, alteration in sequence (PS-CpG-ODN and PS-ApG-ODN) still permitted suppression of aortic sprouts but alteration in backbone (PD-CpG-ODN) lost the ability to suppress the explants irrespective of whether class B ODN1826 or class C ODN2395 (Fig. 2a,b). Both ODN classes when changing sequence of CpG motif (PS-CpG-ODNs or PS-ApG-ODNs) increased sFlt1 production, but only class C PS-CpG-ODNs and PS-ApG-ODNs decreased VEGF production (Fig. 2c,d). These findings indicate that PS-modified backbone is requisite for the anti-angiogenic process regulated through the ODN, but the CpG motif is dispensable. To confirm this, we selected and tested the customised class C ODN2395 in HUVECs, as it reacts with human and mouse cells. Consistent with aortic ring assay, both PS-CpG-ODN2395 and PS-ApG-ODN2395 but not PD-CpG-ODN2395 suppressed tube formation (Supplementary Fig. S2a).

### TLR9 activation is indispensable for suppressing angiogenesis through CpG-ODN *in vivo* but not required to suppress endothelial cells activity

**Figure 3:**
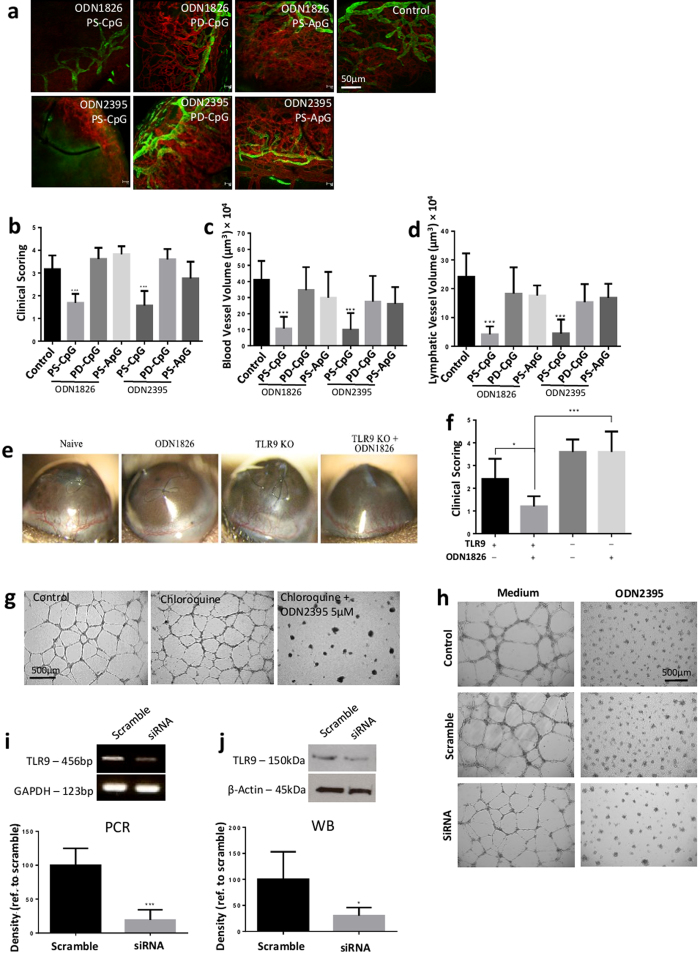
TLR9 activation is indispensable for suppressing angiogenesis through CpG-ODN *in vivo* but not required in suppression of endothelial cell activity. (**a**) Flat mount CD31 (red, 1:100) and LYVE1 (green, 1:200) immunofluorescent staining for sutured cornea 7 days post sub-conjunctival injection with class B and C CpG-ODNs as well as their customized controls (PD-CpG-ODN or PS-ApC-ODN) (n = 18–20 per condition). Images were captured using a confocal laser scanning microscope with x20 magnification lens. Scale bar, 50 μm. (**b–d**) Only PS-CpG-ODNs suppressed the new vessels by reducing the length and volumes of both blood and lymphatic vessels compared to vehicle control. (**e,f**) CpG-ODN1826 did not suppress suture-induced corneal neovascularisation in TLR9 deficient mice compared to water control, whilst ODN1826 significantly suppressed corneal angiogenesis in WT compared to TLR9 deficient mice (n = 6 per condition). (**g**) TLR9 activation inhibitor chloroquine (100 nM) did not regulate HUVECs tube formation. CpG-ODN2395 still had anti-angiogenic effect on HUVECs in the presence of chloroquine (n = 12 per condition). Image capture was performed with Leica DMI6000 microscope (x5). Scale bar, 500 μm. (**h**) CpG-ODN2395 suppressed HUVEC tube formation despite TLR9 siRNA (10nM) transfection (n = 12 per condition). Scale bar: 500 μm. (**I,j**) The efficacy of TLR9 siRNA in HUVECs were examined by conventional PCR and western blot with GAPDH and ß-actin as housekeeping controls. TLR9 production was reduced by 80.8% at transcriptional level and 69.7% at protein level. All the samples were normalized to their β-actin expressions. Data represents means ± SD of relative values vs control from 3 independent experiments. *p < 0.05; **p < 0.005; ***p < 0.0005, statistical analysis was performed with one-way ANOVA with Dunn’s test for multiple comparisons, while unpaired Student’s *t* test and Mann-Whitney test for two individual comparisons.

While stimulation of cells with CpG motifs elicits TLR9 activation, our data suggested that specific motifs may be dispensable for enabling an anti-angiogenic response. Therefore, we wished to determine whether this also applied *in vivo* and whether TLR9 signalling is indispensable for CpG-ODN mediated suppression of angiogenesis. Hence, PS-CpG-, PD-CpG- and PS-ApG-ODNs were tested in the suture induced corneal angiogenesis model. There was no inhibition of corneal neovascularisation when either the CpG motif or the PS backbone were individually substituted, and suppression was only observed in mice that received the PS-CpG-ODN (Fig. 3a–d), suggesting the structural CpG motif is essential to the anti-angiogenic response. The indispensable role of TLR9 signalling *in vivo* was further confirmed by experiments in which administration of ODN1826 in TLR9 deficient mice (TLR9^−/−^) did not suppress vessel growth (Fig. 3e,f).

To understand how TLR9 may regulate endothelial cell responses, we assessed the effect of blocking TLR9 pathways in HUVECs. Chloroquine, a TLR9 activation inhibitor, is an endosomal acidification inhibitor which blocks TLR9 signalling, as acidic pH is a prerequisite for normal enodosomal TLR activation, but it also disrupts the binding of CpG motifs to TLR9 ectodomains^25^. PS-CpG-ODN2395 still suppressed endothelial tube formation despite the presence of chloroquine (Fig. 3g). Furthermore, HUVEC tube formation was also inhibited following PS-CpG-ODN treatment when TLR9 gene expression was silenced using a siRNA approach (Fig. 3h). The efficacy of RNA interference on TLR9 expression was confirmed by PCR and western blot (Fig. 3i,j). The data indicates that intact TLR9 signalling is redundant for ODN-mediated suppression of HUVEC tube formation and the inhibition of endothelial activity. However, TLR9 signaling was indispensable *in vivo*, suggesting that CpG-ODN may inhibit angiogenesis through multiple cell types. We therefore wished to investigate whether the anti-angiogenic effect by CpG-ODNs is pervasive and/or tissue specific, with respect to ocular vasculature.

### CpG-ODN suppresses retinal vessel formation and choroidal neovascularisation

As Class B (ODN1826) and Class C (ODN2395), but not Class A (ODN1585) suppress corneal angiogenesis, we investigated the B and C classes further to determine whether these observations conferred tissue specificity within the eye. During embryonic development, cells derived from the mesodermal and the ectodermal tissues contribute to the formation of the eye, with the neuroectoderm and mesoderm forming the retina and choroid respectively^26^,^27^,^28^. Firstly we examined whether local administration of CpG-ODNs influenced normal retinal vascular development, and secondly, if these could also modulate wound healing responses in the choroid (another vascular compartment of the eye) following laser-photocoagulation^29^.

**Figure 4:**
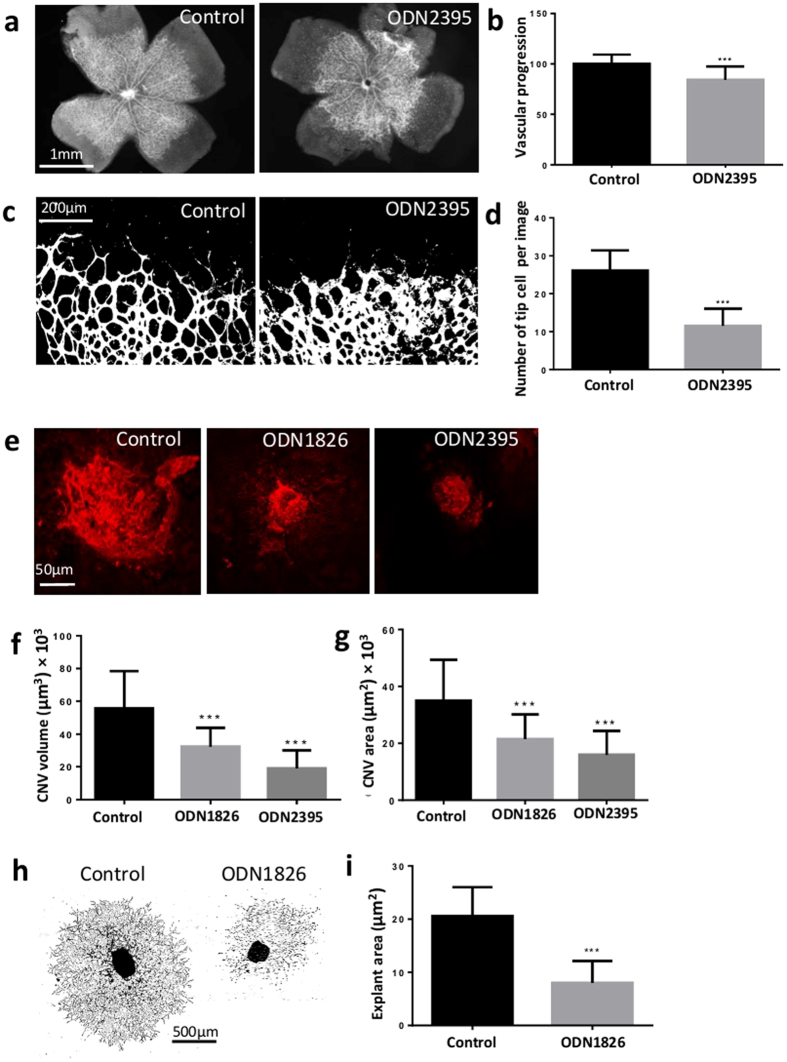
CpG-ODN suppresses retinal vessel formation and choroidal neovascularisation. (**a,b**) Phase contrast photos (x2) of postnatal retina from P4 mice after being injected with either water or CpG-ODN2395 (n = 6–8 per condition). CpG-ODN2395 suppressed the growth of retinal vessels. Images were taken under x10 microscope lens. Scale bar, 1 mm. (**c,d**) Treated retinas from P4 mice were dissected for IB4 staining (1:100) for explant tips counting, CpG-ODN2395 reduced the number of tips in retinas. Scale bar, 200 μm. (**e,g**) Intravitreal injection of CpG-ODN1826 immediately after laser photocoagulation in C57BL/6 mice reduced the area and volume of CNV at day 7 compared to water vehicle mice by evaluation of confocal images of choroid flat mount staining with anti-IB4 antibody (n = 18–22 per condition). Images were captured using a confocal laser scanning microscope with x20 microscope lens. The areas were quantified by Image J and volumes by Volocity. Scale bar, 50 μm. (**h,i**) Phase contrast photos of mouse choroidal sprouts were taken after 7 days culture with or without CpG-ODNs (n = 23–25 per condition). All images were taken under Wide field microscope at x5 magnification for the measurement of sprouting areas. One photo was composed of several x5 images to show the whole area of explants. Scale bar, 500 μm. CpG-ODN1826 suppressed the choroidal sprouts at concentration of 5 μΜ compared to medium control. Data represents means ± SD of relative values vs control from 3 independent experiments. *p < 0.05; **p < 0.005; ***p < 0.0005, statistical analysis was performed with unpaired Student’s *t* test and Mann-Whitney test.

Mouse retinal vessels physiologically develop and reach the peripheral retina during the first week of life (P1-7), which permits assessment of post-natal vascular development. Following intravitreal administration of Class C to the eyes of P4 mice, the length of retinal vessel extension from the optic disc as well as the number of vascular tips were significantly reduced (Fig. 4a–d). Moreover, the area and volume of laser-CNV were also significantly reduced by both Class B and C (Fig. 4e–g). Consistent with these *in vivo* findings, Class B also suppressed the extent of choroidal sprouting and vascular proliferation in the choroidal explant assay (Fig. 4h,i). Together, the data supports that CpG-ODNs modulation of angiogenesis is neither tissue restricted nor reliant on an inciting event, such as wound healing in the laser-CNV model. Therefore, the next step was to understand whether the CpG-ODN mediated effects were indirect, and regulated pericyte and macrophage responses.

### CpG-ODN inhibits endothelial migration and regulates the mobilization of pericytes and macrophages

**Figure 5:**
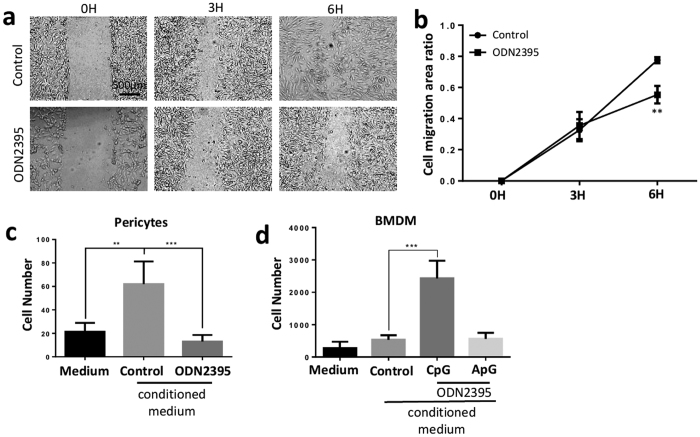
CpG-ODN inhibits endothelial migration and regulates mobilization of pericytes and macrophages. (**a**) Representative phase contrast photos (x5) of gap closure migration assay with 5 μΜ CpG-ODN2395 at 0 h, 3 h and 6 h (n = 16 per condition). Scale bar, 500 μm. (**b**) CpG-ODN2395 at concentration of 5 μΜ delayed the HUVECs migration at 6 h by quantifying the area of the gaps. (**c**) Human pericytes were cultured in upper chamber of transwell with conditioned HUVECs medium or controls in lower chamber (n = 9–12 per condition). After 16 hours culture, the number of migrated pericytes were counted under Leica DMI6000 microscope (x20) after staining the membrane with DAPI. HUVECs culture medium (labelled control) promoted pericytes migration, but additional CpG-ODN2395 treatment suppressed the mobilisation of pericytes. (**d**) Similarly, transwell migration assay with mouse BMDM in upper chamber showed HUVECs culture medium promoted macrophages migration (n = 8–10 per condition). However, in contrast to pericytes, macrophages migration was significantly stimulated by CpG-ODN2395 but not by ApG-ODN2395. Data represents means ± SD of relative values vs control from 3 independent experiments. *p < 0.05; **p < 0.005; ***p < 0.0005, statistical analysis was performed with unpaired Student’s *t* test.

During angiogenesis, the activation of endothelial cells between stalk and tip cells is regulated by Notch and VEGF signalling pathways. The vascular supporting cells, pericytes and macrophages, facilitate the stabilisation and maturation of the angiogenic process. The anti-angiogenic effect on endothelial cells *in vitro* mediated by CpG-ODN alone does not suppress corneal neovascularisation *in vivo*, which led us to investigate how CpG-ODNs may affect the migratory ability of endothelial cells, pericytes and macrophages. Using the gap closure migration assay, ODN2395 significantly delayed the migration of HUVECs (Fig. 5a,b). We performed *in vitro* transwell experiments designed to assess the impact on migratory behaviour, in which human primary pericytes or bone-marrow derived macrophages (BMDM) were seeded in the upper chambers with conditioned medium from ODN2395 stimulated HUVECs. The addition of ODN2395 conditioned HUVEC supernatant suppressed pericytes but accentuated macrophages migration, but the increase in macrophage migration was absent with ApG-ODN2395 conditioned supernatant (Fig. 5c,d). These results demonstrate the ability of CpG-ODN stimulated endothelial cells to restrain mobilization of pericytes yet promote macrophage migration *in vitro*.

### CpG-ODN polarises macrophages toward an M1 phenotype and actively regulates angiogenesis

**Figure 6:**
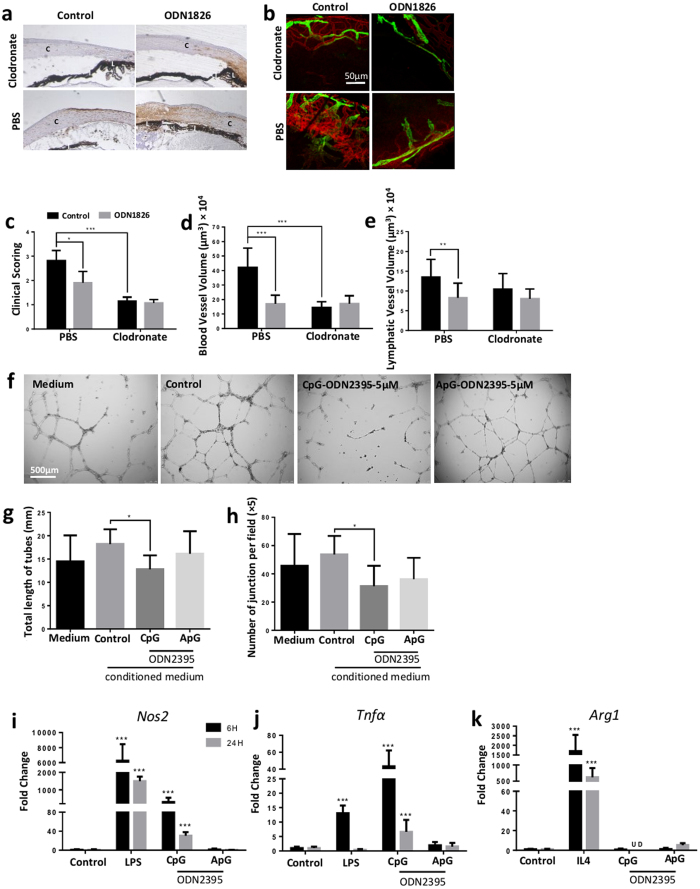
CpG-ODN polarises macrophages to M1 phenotype which actively regulate angiogenesis. (**a**) Immunohistochemistry staining of F4/80 (1:100) showed macrophages influx was increased dramatically by CpG-ODN1826 in sutured cornea (n = 6–8 per condition). Clodronate restrained the significant reduced infiltrating macrophages at the limbus away from mid peripheral cornea of suture site with no recovery after CpG-ODN1826 treatment. Images were taken under x5 microscope lens. C: cornea; I: iris; L: limbus. (**b–e**) Corneal neovascularisation, both blood vessels (red) and lymphatic vessels (green), was significantly reduced in clodronate treated mice compared to PBS treated controls. CpG-ODN1826 did not induce any further reduction of either length or volumes of the neo-vessels (n = 16–18 per condition). Scale bar: 50 μm. (**f,h**) After being stimulated with either CpG-ODN or ApG-ODN for 8 h, the supernatant of macrophages was replaced by fresh medium and leave for another 16 h culture before the supernatant was collected in 1:1 mix with EGM for HUVECs tube formation assay (n = 16 per condition). Supernatant from CpG-ODN2395 but not ApG-ODN2395 treated macrophages reduced both the total length and junction number of tubes compared to control. Scale bar, 500 μm. (**i-k**) BMDMs were cultured for 6 h or 24 h with stimulation of 5 μM CpG-ODN2395, ApG-ODN2395 or medium alone as negative control. LPS (1 μg/mL) and IL4 (20 U/mL) were used as positive controls for M1 and M2 macrophages polarization respectively (n = 12 per condition). CpG-ODN2395 increased the expression of *Nos2* and *Tnfα*, but not *Arg1.* ApG-ODN2395 did not show any effect on these genes expression. UD: undetermined. Data represents means ± SD of relative values vs control from 3 independent experiments. *p < 0.05; **p < 0.005; ***p < 0.0005, statistical analysis was performed with one-way ANOVA with Dunn’s test for multiple comparisons, while unpaired Student’s *t* test and Mann-Whitney test for two individual comparisons.

The current data shows that CpG-ODNs can enhance the migration of macrophages, which correlates with the previous observation of an increased number of infiltrating macrophages in sutured-cornea following CpG-ODN1826 administration^12^. The intrinsic plasticity of macrophages facilitates different functional responses depending on signals received in the tissue microenvironment^30^,^31^, and increasing evidence support the active regulatory role of macrophages in angiogenic processes^32^. To further interrogate whether the CpG-ODN mediated suppression of angiogenesis was dependent on macrophage involvement, we depleted macrophages both systemically and locally by administration of clodronate liposomes. The efficacy of macrophage depletion systemically and locally (cornea and surrounding tissue) by clodronate was examined by immunohistochemistry staining of spleen and eye (Fig. 6a and Supplementary Fig. S3a,b). In the control (PBS liposomes) treated cornea, there was influx of myeloid cells with predominantly F4/80+ macrophages from limbus to mid-peripheral cornea post suturing. In contrast, only minimal infiltrating F4/80^+^ and CD11b^+^ cells were present in clodronate treated cornea (Fig. 6a and Supplementary Fig. S3b). ODN1826 increased the number of corneal infiltrating macrophages in both PBS and clodronate treated mice, however, infiltration was still considerably lower in clodronate treated mice. Depletion of macrophages by clodronate was accompanied by reduction of corneal neovascularisation, although additional CpG-ODN did not augment this anti-angiogenic effect (Fig. 6b–e).

If macrophages are required in corneal neovascularisation and CpG-ODN augmented macrophages infiltration, we wished to further discern how CpG-ODN stimulated macrophages may regulate endothelial cell responses. The pre-conditioned culture supernatant from the macrophage cultures was added to HUVECs to assess tube formation. The excess of CpG-ODN was removed by changing to fresh medium after 8 h stimulation to minimize the direct effect of CpG-ODN. Only supernatants from CpG-ODN pre-conditioned macrophages were able to suppress HUVEC tubes (Fig. 6f–h). This suggested that CpG-ODN can stimulate macrophages to elicit mediators that directly modulate the angiogenic activity of endothelial cells.

We next explored whether CpG-ODN could polarise macrophages towards either an M1 or M2 phenotype. Accordingly, the responses of BMDM stimulated with CpG-ODN2395, ApG-ODN2395 or medium control for 6 h and 24 h were analysed. LPS and IL-4 were included as stimuli controls for M1 and M2 polarization respectively. CpG-ODN increased the mRNA expression of *Nos2* and *Tnfα*, signatures of M1 activation^33^, but did not induce *Arg1* expression of M2 activation (Fig. 6i–k)^33^. The control ApG-ODN did not upregulate any *Nos2, Tnfα* or *Arg1* expression. In addition, none of the ODN treatments regulated transcriptional expression or protein levels of *sFlt1* or *Vegf* in macrophages (Supplementary Fig. S3c,d). These results support that CpG-ODN polarises macrophage towards an M1 phenotype.

## Discussion

The role of TLR9 in inflammation has long and widely been recognized^34^,^35^. More recently increasing studies haav demonstrated its role in angiogenesis^21^,^36^. We have previously shown that Class B CpG-ODN1826 suppresses corneal neovascularisation^12^. We now demonstrate the direct anti-angiogenic effect of CpG-ODNs on vascular endothelial cells independent of TLR9 signalling. TLR9 is indispensable for macrophage regulation of angiogenesis, as the effect is lost in the absence of CpG motif or in TLR9 deficient mice. The significance of the anti-angiogenic effect on multiple ocular disease models is the translational potential towards adjunct therapy for blinding angiogenic ocular disorders. Such therapeutic potential is further supported by the current assessment of TLR9 agonist CpG-ODN as vaccine adjuvants as a strategy in cancer research^37^. The current notion is that CpG-ODNs augment specific immune responses through TLR9 mediated NF-κB activation, as well as CpG-ODN itself exhibits anti-tumour activity via reduction of VEGF and HIF-1α^38^. Notwithstanding, it is evident from the data presented here that CpG-ODNs have wider actions that might offer exciting therapeutic exploitation, especially for ocular disorders where abnormal neovascularisation leads to blindness.

The anti-angiogenic effect of CpG-ODN within the eye is tissue nonspecific and pervasive as we observed attenuation of new vascular growth in a wide range of *in vitro* and *in vivo* models. Furthermore, all three classes of CpG-ODNs exhibited anti-angiogenic capacity, and predominantly through upregulation of sFlt-1. In aortic ring sprout assay, class A CpG-ODN suppressed VEGF secretion and class B stimulated sFlt1, whereas class C regulated both VEGF and sFlt1 (Fig. 1c,d). sFlt1 is a potent anti-angiogenic protein with high affinity to VEGF, and is highly upregulated by both class B and C ODNs corroborating the reduced VEGF secretion in aortic ring culture. Only class B and class C suppress corneal angiogenesis *in vivo*, which might be explained by the instability of the backbone of class A CpG-ODN compared to the other two classes^14^. To elucidate the mechanism by which CpG-ODNs may modulate angiogenesis, we demonstrate the influence of the ODN backbone. As the classes of ODNs not only differ in their number and location of CpG, but also the type of backbone, we customised class B and C ODNs with single nucleotide or backbone substitutions to the control ODNs. Both PS-ODNs with CpG or ApG, but not PD-ODN, displayed anti-angiogenic responses in aortic ring assay, suggesting that the PS backbone is responsible for inhibition of the aortic vascular sprouts, and sequence specificity is not requisite. We also observed reduced tube formation in HUVECs following ODN treatment, and again this was independent of either the ODN sequences or CpG motifs (Fig. 1e and Supplementary Fig. S2). It is recognized that in antisense biotechnology, one antisense oligonucleotide in theory may degrade or block its target RNAs, but that oligonucleotides may also have biological effects that are not attributable to their sequences^39^. Therefore, their effect on cellular processes can be due to non-specific binding of oligonucleotides to proteins, or the reagents used to deliver the oligonucleotides. Many of the non-sequence-specific effects may be the result of the increased affinity of phosphorothioate ODN for binding proteins^40^. Phosphorothioate thymidine homopolymers, in contrast to other homopolymers, can induce the stimulation of human B cells or murine splenocytes^41^,^42^. The number of thymidine nucleobases in non-CpG phosphorothioate ODN, as well as the length of such ODN, directly relate to the magnitude of their stimulatory effects^43^,^44^. Although phosphodiester (PD) is the natural backbone present in bacterial and viral DNAs, its use in synthetic ODNs is limited due to rapid degradation by the intracellular endonucleases and exonucleases^45^. In contrast, phosphothioates (PS) are widely used because of their nuclease stability and efficient cellular uptake. Whilst an off-target effect on endothelial cells may contribute to anti-angiogeneic effects, it is not sufficient alone without TLR9 activation *in vivo*. To address the direct effect of CpG-ODN on endothelial cells, further data may identify the potential mechanisms driving off-target effects.

Angiogenesis requires VEGF-VEGFR2 signalling pathways. During sprouting, Dll4-Notch1 signalling selects the endothelial tip cells to lead the outgrowth of sprouts towards VEGF gradients^46^. To identify the anti-angiogenic effectors stimulated by ODNs in HUVECs and cornea, *Vegf, Dll4, Notch1,* and *sFlt1* mRNAs were quantified. Among these, *Dll4* and *Notch1* were upregulated at 6 h in HUVECs (Supplementary Fig. S4). Dll4 is expressed in tip cells and acts on stalk cells to suppress stalk to tip cell conversion. Dll4 also binds to Notch receptors on neighbouring cells, and in turn downregulates VEGFR2 inhibiting endothelial cell proliferation^47^,^48^. The increased expression of *Dll4* and *Notch1* suggests that CpG-ODN maintains HUVECs at stalk cell status, a finding that is supported by the observation of unchanged level of *Vegf* and *sFlt1* (Supplementary Fig. S4). Although no significant change of *Dll4*, *Notch1, Vegf* or *sFlt1* mRNA expression was found in corneas after CpG ODN (Supplementary Fig. S1e), it is possible that these signals from tip cells localised at limbus in the multicellular responses were too weak to be detected *in vivo*. We also observed delayed development of retinal vasculature and reduced number of vascular tips by CpG-ODN during the first post-natal week in mice (Fig. 4a–d). However, the exact mechanism remains unclear as to how the CpG-ODNs restrain stalk cell status from subsequent tip cell formation.

If the TLR9 binding molecule CpG motif is dispensable for anti-angiogenic effect on vascular endothelial cells, it is reasonable to assume that TLR9 activation is also non-essential. *In vitro* results indicate that perturbing TLR9 signalling in endothelial cells with either chloroquine or siRNA does not impact HUVEC microtube formation by ODNs (Fig. 3g,h). The exact binding site of CpG-ODN in endothelial cells remains unclear. It has been deomonstrated that TLR9 is retained in the endoplasmic reticulum prior to stimulation and TLR9 bypasses the Golgi complex before translocation to endolysosomes where binding to CpG-ODN occurs^49^. Nevertheless, we observed cytoplasmic distribution of ODN2395 within endothelial cells (Supplementary Fig. S5). However, in the corneal neovascularisation model treatment with CpG-ODNs attenuated angiogenesis, indicating the participation of an elicited innate immune response by CpG in the anti-angiogenic process. To this end, we showed that the effect of suppressing corneal neovascularisation by CpG-ODN was diminished in TLR9^−/−^ mice (Fig. 3e,f). The data confirms that the anti-angiogenic effect, at least in the cornea, is both PS-backbone and TLR9 dependent. Although the role of endothelial cells is essential in angiogenesis, there are multiple cell types taking part in the process^50^,^51^, The TLR9-independent effect on endothelial cells may contribute to but is not sufficient for angiogenic suppression without TLR9 activation in the corneal angiogenesis model.

Stabilisation and maturation of the vascular sprout during new vessel formation is accompanied by the recruitment of pericytes and macrophages, which involves cellular cross talk in an autocrine and/or paracrine manner^52^,^53^. Not surprisingly perhaps, but nevertheless not recognised previously, was the significant reduction in pericyte migration demonstrated *in vitro* following CpG treatment (Fig. 5c). Many studies detail the role of macrophages in regulating angiogenesis^54^. However, we show profound preclinical effects of CpG-ODN despite the influence of CpG-ODN immune activation^34^, and as we show, with a macrophage M1 polarization and attenuation of endothelial responses. In the absence of macrophages, angiogenesis was suppressed but not augmented by CpG-ODN (Fig. 6b–e). Although free clodronate inhibits angiogenesis by suppressing endothelial cell proliferation^55^, liposome-encapsulated clodronate has no effect on endothelial or smooth muscle cells^56^. The paradigm of co-existence of inflammation and anti-angiogenesis elicited by CpG-ODN can be favourable in treating tumours and now with this data, ocular vascular diseases. Here we showed that the CpG-ODN stimulated macrophages inhibit vascular endothelial cell (HUVEC) tube formation (Fig. 6f–h), with further demonstration that CpG, as an immune-stimulator, actives TLR9 in macrophages to initiate inflammation, and polarises macrophages to an M1 anti-angiogenic phenotype^57^,^58^ (Fig. 6i–k). This data also converges with the growing evidence that M1 tumour associated macrophages are involved in the inhibition of angiogenesis as well as anti-tumour immunity in addition to their traditional pro-inflammation role^59^,^60^. Nevertheless, further experiments are required to confirm the interplay of macrophages and endothelial cells *in vivo* in the presence of CpG-ODN.

In summary, we demonstrate that all three classes of CpG-ODNs have an anti-angiogenic effect on endothelial cells, which requires backbone phosphorothioate but not TLR9 signalling. In contrast, CpG motifs and TLR9 activation are necessary for macrophages polarization to M1 phenotype and further suppression of endothelial cell activity to augment the anti-angiogenic responses. Therefore, the suppression of angiogenesis mediated by CpG-ODNs modulates both endothelial cells and macrophages through distinct pathways, and provides potential therapeutic application in ocular vascular diseases.

## Methods

### Cell culture and reagents

Primary Human Umbilical Vein Endothelial Cells (HUVEC) (PromoCell, Heidelberg, Germany) were cultured in endothelial cell growth medium (EGM, Promocell) to passage 4 or 5 before being treated with various CpG-ODNs (Supplementary Table S1) (TIB Molbiol, Berlin, Germany). Pericytes were immuno-sorted from saphenous vein leftovers of coronary artery bypass graft surgery patients as described previously^61^,^62^. Bone marrow derived macrophages (BMDM) were prepared from 4–6 weeks old C57BL/6 mice as described before^63^. BMDM cultures were stimulated with IL-4 (20 U/mL) (PeproTech, UK), LPS (1 μg/mL) (Sigma) and CGS-21680 (10 nM) (Sigma). Studies on cells from human subjects complied with the principles stated in the “Declaration of Helsinki” and were covered by approval (06/Q2001/197) from the Bath Research Ethics Committee.

### Animals

Adult (6–10 weeks) B10.RIII, C57BL/6 and B6LY5 mice were bred and housed in University of Bristol. Postnatal (P) 4 C57BL/6 mice were bred and housed in UCL central lab. Balb/c mice, aged 6–8 weeks, were purchased from Harlan UK Limited (Blackthorn, UK). TLR9 deficient mice backcrossed to C57BL/6 background, were provided and bred in Zhongshan Ophthalmic Center of Sun Yat-Sen University. The strain of neonatal mice was C57BL/6. All the animal experiments were undertaken in both sexes. All procedures were conducted under the regulation of the United Kingdom Home Office Animals (Scientific Procedures) Act 1986, and were in compliance with the Association for Research in Vision and Ophthalmology (ARVO) statement for the use of animals in ophthalmic and vision research. The methods were carried out in accordance with the approved University of Bristol institutional guidelines and all experimental protocols under Home Office Project Licence 30/3045 and 30/3281 were approved by the University of Bristol Ethical Review Group.

### siRNA transfection in HUVECs

A pool of 3 independent small interfering RNA (siRNA) targeting different sequences of TLR9 mRNA and their scramble sequences were purchased from Thermo Scientific. HUVECs were transfected with siRNA (10 nM) using lipofectamine RNAiMAX (Life Technologies, Invitrogen) according to manufacturer’s protocol. The knock-down efficiency was detected by conventional PCR (TLR9, forward: 5-CTACAACCGCATCGTCAAAC-3; reverse: 5-ATCGAGTGAGCGGAAGAAGA-3) and western blot (anti-human TLR9, Abcam, Cambridge, UK).

### ELISA

The protein expression of VEGF and sFlt-1 in conditioned media from aortic ring cultures were determined by mouse VEGF and sFlt-1 ELISA kits (R&D Systems, Minneapolis, MN, USA) according to the manufacturer’s protocols. The absorbance was measured at 450 nm on a microplate reader and VEGF and sFlt1 concentrations were calculated using linear regression analysis.

### Quantitative RT-PCR

Total mRNAs were purified from HUVECs, mouse corneas or BMDM using the RNeasy Mini Kit (Qiagen, Hamburg, Germany) as described by the manufacturer protocol. The mRNAs were reverse-transcribed using the Reverse Transcription System (Promega, Wisconsin, USA). The two-step qRT-PCR SYBR Green method was used to test the gene expressions as listed in supplementary table S2, which were measured in the same sample and relative ratios of fluorescent intensities of products from each treatment group were calculated by stimuli using the 2^−ΔΔCt^ method^64^.

### Western blotting

Protein was extracted from TLR9 siRNA or scrambled RNA transfected HUVECs by Cellytic MT buffer (Sigma) according to the manufacture’s protocol. The concentration of protein was determined using BCA kit (Thermo Scientific). Membranes were incubated with HRP-conjugated secondary antibody (Cell signalling, MA, USA). The signals were developed with ECL reagent (Sigma) and captured by an electronic imaging system (Konica Minolta). β-actin (Cell signalling) was used as house-keeping control. Each samples was loaded as 10 μg protein in 40 μL. The quantification was achieved by normalizing each sample to its β-actin expression.

### HUVEC tube formation and migration assay

P5 HUVECs were seeded on Matrigel (BD Biosciences) in endothelial cell basic medium (EBM) with 100 ng/mL VEGF (Sigma) and with or without treatments. Different doses of CpG-ODNs or their customized controls were used to treat HUVEC for 24 hours before assessment of tube formation. Chloroquine (InvivoGen, France), a lysosomotropic agent that prevents endosomal acidification^65^, was used to treat HUVECs to inhibit TLR9 activation with the stimulation of CpG-ODNs. For the conditioned medium from macrophages, CpG-ODN or ApG-ODN was used to stimulate cells for 8 hours, before the supernatant was replaced by fresh medium without stimulation. Following further incubation for 16 h in fresh medium, the culture supernatant was collected before adding to HUVECs to access tube formation. The conditioned medium was mixed with HUVECs culture medium as 1:1 ratio for each well. HUVECs were seeded in culture-inserts in EBM (Ibidi, Germany). After incubation at 37 °C overnight, all  the inserts were removed before adding fresh EBM with or without ODN treatments. Phase contrast photos were taken post treatment by Wide field microscope (Leica DMI6000). The whole area of gaps was quantified by ImageJ1.46r (National Institutes of Health, USA) and ratio of gap closure was calculated by division of 0 h gap for each time point.

### Transwell migration assay

HUVECs were cultured in EBM with or without ODN treatment for 24 hours before the supernatant was transferred to the lower chamber of transwell (Corning, UK), while the upper chamber was seeded with human pericytes or BMDM for 16 hours. EBM medium alone was included in lower chamber as negative control. The membranes of the upper chamber were fixed with 4% paraformaldehyde followed by staining with DAPI (4′, 6-diamidino-2-phenylindole) for cell counts. Five fields of photos were randomly taken under x20 microscope for each down-side membrane.

### Aortic ring assay and choroidal explant assay

Aortic rings were prepared from wild-type B10.RIII mice aged 4–6 weeks as previously described^66^. Aortic rings were embedded in collagen gel (Millipore) before being treated with different doses of CpG-ODNs, their customized controls or medium alone from day 0. After 7 days of treatment, supernatant was collected to determine VEGF and sFlt1 expression by ELISA, while aortic rings were fixed for flat mount immunofluorescence. The total number of sprouts were counted under confocal microscope. Mouse peripheral choroid-scleral complex was dissected from B10.RIII mice aged 6-8 weeks and cut into approximately 1 × 1 mm pieces before being embedded in Matrigel (BD Biosciences) as described before^67^. Phase contrast photos of explants were taken on day 7, and the sprouting areas were quantified using ImageJ 1.46r (National Institutes of Health).

### Suture induced corneal neovascularization model

Balb/c mice or TLR9^−/−^ mice received 3 interrupted 11-0 Nylon sutures (Mani, Japan) at 1 mm from limbus after anesthesia by intraperitoneal (i.p.) injection of Vetelar (ketamine hydrochloride 100 mg/mL, Pfizer, UK) and Rompun (xylazine hydrochloride 20 mg/mL, Bayer, UK) mixed with sterile water in the ratio 0.6:1:84. Sub-conjunctival administration of 3 μL water or ODNs was performed on alternate days. The length and volume of outgrowing corneal neovessels were scored under surgical and confocal laser scanning microscope on day 7 with scoring system (Supplementary Table S3).

### Macrophage depletion

Macrophage depletion in B6LY5 mice was achieved by administration of clodronated-liposomes^68^ (ClodronatedLiposomes.com, Amsterdam, Netherlands). For systemic macrophage depletion, 1 mg/200 μL of clodronate or PBS liposomes was administrated intraperitoneally; whereas for local depletion, 0.05 mg/10 μL clodronate or PBS liposomes were delivered via sub-conjunctival injection on days 3 and 6. The efficacy of macrophage depletion by clodronate was confirmed by immunohistochemistry staining for F4/80^+^ and CD11b in spleen and eye cryosections. Corneal suturing was performed at day 7 on the clodronate treated mice.

### Vascularisation of neonatal retina

Mice were given intravitreal injection of either water or CpG-ODN (0.4 μg/0.25 μL) at postnatal day (P) 4. After 24 h, mice were sacrificed and retinas were dissected for immunofluorescence staining with anti-IB4 (1:100, Sigma). Representative images demonstrate the typical vascular phenotype observed in at least eight retinas. Image quantifications were performed using ImageJ. The number of filopodial extensions were quantified at the retina angiogenic front in a minimum of 12 fields (sized 1256 μm × 957 μm). Vascular progression was measured by defining a straight line from optic disc to the angiogenic front for each retina quadrant in a low magnification stereomicroscope pictures. There were 12 measurements for each retina.

### Laser-induced choroidal neovascularisation (CNV) Model

The pupils of B10.RIII mice were dilated using topical 1% tropicamide (0.5% w/v, Bausch & Lomb, Aubenas, France), before induction of anesthesia. Four laser spots were introduced in each eye on the posterior retina using OculightSlx Krypton Red Laser system (power 200 mW, duration 75 ms, spot size 75 μm). Immediately following laser, local administration of Class B CpG-ODN1826 (500 μM in 2 uL dH_2_O) or vehicle control was performed by intravitreal injection. Mice were sacrificed on day 7 post laser. The eyes were collected for immunostaining with biotinylated isolectin IB4 antibody (Sigma), followed by staining with Streptavidin-Rhodamine Red X (1:300, Jackson Immuno Research Laboratories).

### Immunohistochemistry and immunofluorescence

Dissected corneas were fixed in 2% paraformaldehyde over night at 4 °C. The cornea whole mounts were co-stained with anti CD31- APC (BD Biosciences) and purified rabbit anti mouse LYVE-1 (Abcam) antibodies, followed by secondary goat anti rabbit Alexa Fluor 488 (Invitrogen). The volume of blood and lymphatic vessels was quantified using Volocity version 6.2.1 software (PerkinElmer, Massachusetts, USA). Mouse aortic rings were stained using rat anti-CD31-APC. All Images were captured by Leica SP5-AOBS confocal laser microscope. Mice eyes were snap frozen in OCT (optical cutting temperature compound, Thermo, Waltham, MA, USA). Cryosections were fixed in acetone and immunostained with rat anti-mouse biotinylated CD11b (at dilution 1:100, BD Phamingen), and F4/80 (at dilution 1:100, BD Biosciences) antibodies in conjunction with the HRP conjugated StrepABC kit (Vector Labs, Burlingame, CA) and DAB substrate kit (Vector Labs). Mouse IgG isotype was used as a negative control at dilution 1:100 (BD Biosciences).

### Statistics

Results are therefore presented as means ± standard deviation (S.D.). Comparisons of two individual experimental groups were performed by unpaired Student’s *t* test and Mann-Whitney test. For multiple comparisons, nonparametric analysis was performed using one way ANOVA test with Dunn’s test. All the analysis was performed using GraphPad Prism 6 (GraphPad Software, version 6.01, La Jolla, USA). Two-tailed tests were used throughout. The significant differences were considered at *P* ≤ 0.05.

## Additional Information

**How to cite this article**: Wu, J. *et al*. Pleiotropic action of CpG-ODN on endothelium and macrophages attenuates angiogenesis through distinct pathways. *Sci. Rep.* **6**, 31873; doi: 10.1038/srep31873 (2016).

## Supplementary Material

Supplementary Information
