# Supplementary material for: Pleiotropic action of CpG-ODN on endothelium and macrophages attenuates angiogenesis through distinct pathways
Source: Sci Rep. 2016 Aug 25;6:31873. doi: 10.1038/srep31873 (PMC4997267; doi:10.1038/srep31873)
Supplement: Supplementary Information [file srep31873-s1.pdf]

Supplementary Information

Pleiotropic action of CpG-ODN on endothelium and macrophages attenuates angiogenesis through distinct pathways

Jiahui Wu, Wenru Su, Michael B. Powner, Jian Liu, David A. Copland, Marcus Fruttiger, Paolo Maddedu, Andrew D. Dick, Lei Liu

Supplementary Tables

Table S1. List of CpG-ODNs

	Class	Backbone	Sequence (5'-3')
PS-CpG-ODN2216	A	PS	ggGGGACGA:TCGTCgggggg
PS-GpC-ODN2216	control	PS	ggGGGA**GC**A:T**GC**TGgggggg
PS-CpG-ODN1826	B	PS	TCCATgACgTTCCTgACgTT
PD-CpG-ODN1826	B	PD	TCCATgACgTTCCTgACgTT
PS-ApG-ODN1826	control	PS	TCCATgA**A**gTTCCTgA**A**gTT
PS-CpG-ODN2395	C	PS	TCgTCgTTTTCggCgCgCgCCg
PD-CpG-ODN2395	C	PD	TCgTCgTTTTCggCgCgCgCCg
PS-ApG-ODN2395	control	PS	T**A**gT**A**gTTTT**A**gg**A**g**A**g**A**g**AA**g

Table S2. List of sequences

Gene name	Sequences
mVegf	F 5'-AGCAGAAGTCCCATGAAGTGA-3'
	Rv 5'-ATGTCCACCAGGGTCTCAAT-3'
msFlt1	F 5'-GCCGGGCCTTCAATAAAATA-3'
	Rv 5'-CTTTTTGCCGCAGTGCTC-3'
mGapdh	F 5'-TTCACCACCATGGAGAAGGC-3'
	Rv 5'-GGCATGGACTGTGGTCATGA-3'
hVegf	F 5'-GAAGTGGTGAAGTTCATGGATGT-3'
	Rv 5'-TGGAAGATGTCCACCAGGGTC-3'
hsFlt1	F 5'-GGCTGTTTTCTCTCGGATCTC-3'
	Rv 5'-CATCTCCTCCGAGCCTGA AAG-3'
hDll4	F 5'-GCGGGGTACCTTCTCGCTCATCAT C-3'
	Rv 5'-GCCTCCCCAGCCCTCATCACAAGTA-3'
hNotch1	F 5'-CAGGCAATCCGAGGACTATG-3'
	Rv 5'-CAGGCGTGTTGTTCTCACAG-3'
hTie2	F 5'-CACAAGTACCCTACTGCGGGATGACTTGTG-3'
	Rv 5'-TTCTCCCGCCAGCATTGTCT-3'
hGapdh	F 5'-GGTGTGAACCATGAGAAGTATGA-3'
	Rv 5'-GAGTCCTTCCACGATACCAAAG-3'

Table S3. Corneal neovascularisation scoring

Score	Vessel Length
0	No Vessel
1	0-0.25 mm
2	0.25-0.5 mm
3	0.5-0.75 mm
4	0.75-1.0 mm

Supplementary Figure S1

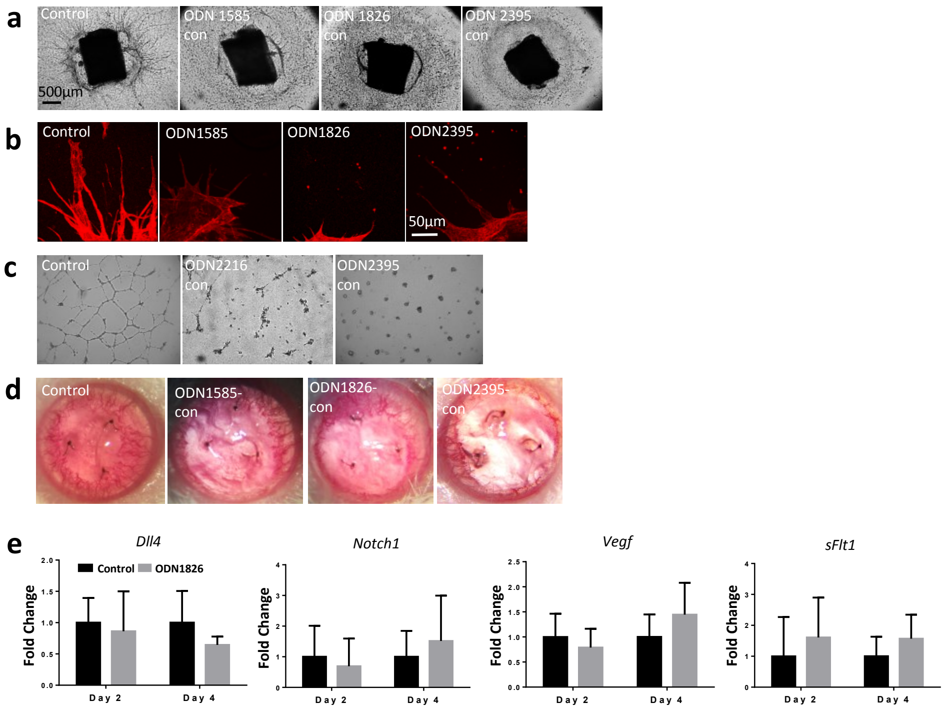

Figure S1: (a) Aortic rings were seeded in collagen gel with stimulation of non-CpG controls of three classes of CpG-ODNs at the dose of 5 µM. All non-CpG-ODN controls suppressed the angiogenic sprouts of aortic rings. (b) Aortic rings were stained for anti-CD31-APC (red) and imaged under ×20 magnification for counting of explants. (c) Both of the CpG-ODN controls (non-CpG-ODN2216 or non-CpG-ODN2395) at concentration of 5 µM inhibited HUVECs tube formation after 24 h. (d) None of the non-CpG-ODNs inhibited corneal angiogenesis. (e) Sutured corneas were dissected from either day 2 or day 4 post sub-conjunctival administration of CpG-ODN1826 or water (n = 12-14 per condition). There was no significant change in the expression of *Dll4, Notch1, Vegf* and *sFlt1* in corneas between ODN1826 and water control. Data represents means ± SD of relative values vs control from 3 independent experiments. Statistical analysis was performed with unpaired Student's *t* test and Mann-Whitney test.

Supplementary Figure S2

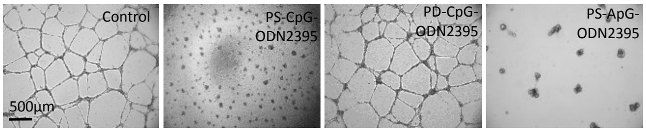

Figure S2: P4 HUVECs were seeded on Matrigel with stimulation of CpG-ODN2395 or its customized controls. Phase contrast photos were taken after 24h incubation. Both PS-CpG-ODN and PS-ApG-ODN but not PD-CpG-ODN suppressed HUVEC tube formation. Scale bar: 500 μm.

Supplementary Figure S3

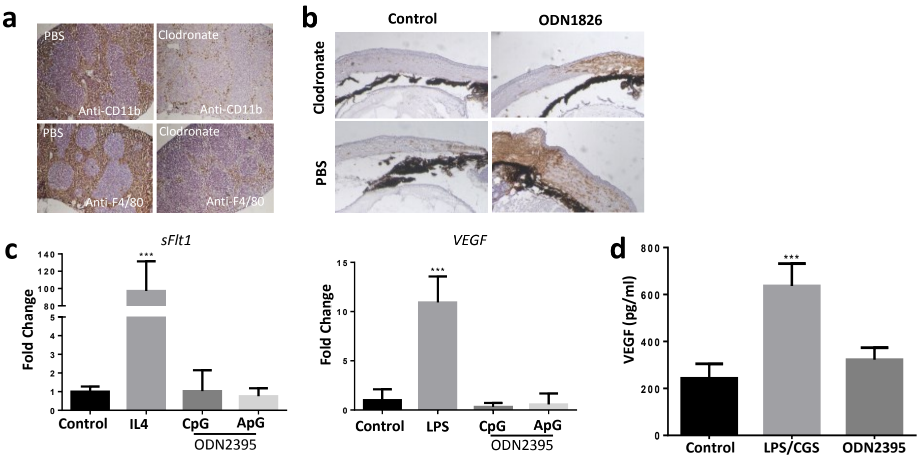

Figure S3: (a) The spleen sections from clodronate or PBS control treated mice were stained for anti-CD11b (1:100) and anti-F4/80 (1:100) for examining the efficiency of systemically macrophages depletion by clodronate. (b) The sutured eyes were collected from clodronate or its PBS control treated mice after 7 days post sub-conjunctival administration of either water or CpG-ODN1826. The sections of eyes were stained for anti-CD11b for efficiency of local macrophages depletion. (c) BMDMs were cultured with stimulation of 5 µM CpG-ODN2395 or ApG-ODN2395 for 6 h or 24 h. IL4 (20 U/mL) and LPS (1 µg/mL) were used as the positive control for sFlt1 and VEGF expression respectively. The expression of *sFlt1* and *Vegf* were not significantly regulated by either CpG-ODN or ApG-ODN. (d) BMDM were cultured for 48 h with stimulation of medium alone, LPS/CGS (1 µg/mL/10 nM) or CpG-ODN2395 (5 µM). The VEGF level in supernatant was increased by LPS/CGS but not CpG-ODN2395 compared to medium alone (n = 12 per condition). Data represents means ± SD of relative values vs control from 3 independent experiments. ***$p < 0.0005$, statistical analysis was performed with one-way ANOVA with Dunn's test for multiple comparisons.

Supplementary Figure S4

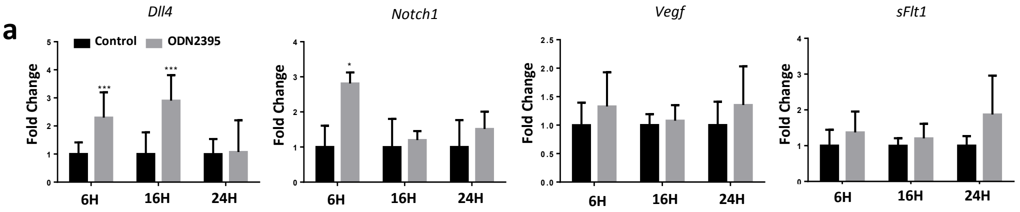

Figure S4: HUVECs were cultured for 6 h, 16 h or 24 h with CpG-ODN2395 (5 µM) or medium alone as control. CpG-ODN2395 significantly up-regulated expression of *Dll4* at 6 h and 16 h and *Notch1* at 6 h compared to control (n = 12 per condition). The expression of *Vegf* and *sFlt1* was not significantly regulated at any time point. Data represents means ± SD of relative values vs control from 3 independent experiments. *$p < 0.05$; ***$p < 0.0005$, statistical analysis was performed with unpaired Student's *t* test and Mann-Whitney test for two individual comparisons.

Supplementary Figure S5

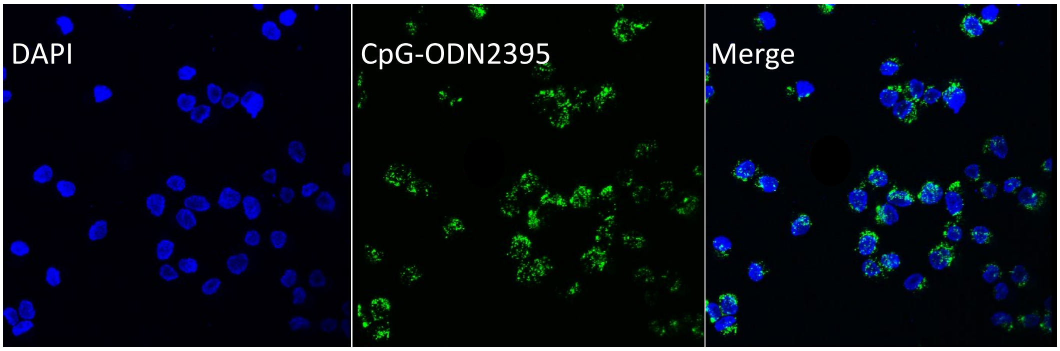

Figure S5: HUVECs were cultured for 6 h with CpG-ODN2395 conjugated to FITC (5 μM). Cells were fixed and followed by DAPI staining before Confocal scanning (n = 6 per condition). Photos represent that CpG-ODN2395 has entered cytoplasm after 6 hours stimulation.
